# Children and Young People’s Improving Access to Psychological Therapies: inspiring innovation or more of the same?^†^

**DOI:** 10.1192/pb.bp.114.047118

**Published:** 2015-04

**Authors:** Sami Timimi

**Affiliations:** 1 Horizon Centre, Lincoln

## Abstract

In 2007 the UK Government announced a substantial expansion of funding for psychological therapies for those presenting with common mental health problems. This ‘Improving Access to Psychological Therapies’ (IAPT) project was widely welcomed, however, evidence backed, economic, and conceptual critiques were voiced from the start and the project remains controversial. In 2011, the UK government announced it was extending the IAPT project to encompass services for children and young people with the aim of ‘transforming’ the way mental health services are delivered to them. Here I critically reflect on the problems associated first with IAPT and then with CYP-IAPT and ponder whether CYP-IAPT is significantly different to the problematic adult IAPT project or more of the same.

## Improving Access to Psychological Therapies (IAPT)

For those who believe that psychological therapies help people, there was much to celebrate in a plan to dramatically increase access to these therapies and decrease waiting times, allowing more people with common mental health problems, such as anxiety and depression, to recover. However, from the start IAPT has had a mixed reputation in professional circles, although this has not yet affected its continued expansion. Why did it become so controversial? One of the main reasons has been the ‘fetishisation’ of certain therapeutic modalities (particularly cognitive–behavioural therapy (CBT)) resulting from an adherence to a primarily technical understanding of the nature of mental health problems and their solutions. This stance marginalises the evidence base that points to the limitations of the technical paradigm, sets up an artificial hierarchy of desirability and efficacy for psychotherapies (and therefore psychotherapists), encourages medicalisation and leads to claims about efficiency that has not been matched by the available evidence.

The technical model assumes that: mental health problems arise from faulty mechanisms or processes of some sort involving abnormal physiological or psychological events occurring within the individual; that these mechanisms or processes can be modelled in causal terms and are not context-dependent; and that treatments can be designed and delivered in a manner that is independent of relationships, contexts and values.^1^ Thus, like National Institute fore Health and Care Excellence (NICE) guidelines, IAPT’s foundational paradigm follows a pathway that assumes that a correct diagnosis (of mild to moderate depression for example) will enable the correct choice of a technical intervention to be made. In this linear formatting, process-driven protocols are central and relational and other contextual issues are of secondary importance – issues to be negotiated to enable satisfactory adherence with the required ‘correct’ treatment.

From a purely evidence-based point of view, this technical model for delivering mental healthcare has little to support it. Whereas factors outside of therapy (the real-life challenges and histories) have by far the biggest impact on outcomes, within treatment, the factor that has the biggest impact on outcomes is the therapeutic alliance (as rated by the patient) with matching treatment model to diagnosis having a small to insignificant impact.^2,3^ This relationship between the alliance and outcome seems remarkably robust across treatment modalities and clinical presentations.^4^ The search for the ‘active ingredients’ of a psychological therapy is anyway likely to be doomed to failure because it depends upon the false assumption that such ingredients are delivered by therapists in a uniform manner regardless of the state, requirements and input of the patient.^5^ After all, although the utterances of a patient may not have a direct impact on the chemical activity of a drug, they will in the two-way process of a well-delivered talking therapy. Thus we should not be surprised that attempts to find the active ingredients of, for example, CBT have failed, as studies have shown that most of the specific features of CBT can be dispensed with, without adversely affecting outcomes.^6,7^ Countless reviews and meta-analyses have found that no clear pattern of superiority for any one treatment model has emerged,^8,9^ a finding that extends into evaluations of NHS psychotherapy services, where non-specific factors such as the therapeutic relationship accounts for the variance in outcomes rather than the therapeutic model used.^10^

As a result, the IAPT process, which insists on one or two modalities, ends up limiting choice for patients, despite the lack of evidence supporting such a stance. It is possible, of course, that choice had to be sacrificed to maximise the efficiency that may come from standardisation, but the evidence here provides no encouragement either.

Not surprisingly, the IAPT project leaders have reported favourable results being delivered by their services;^11,12^ however, their reports do not include comparisons with the costs and outcomes achieved by non-IAPT services. The first independent evaluation of the initial IAPT pilot sites found little difference between the IAPT sites and comparator services. What differences there were in outcomes were not significant 4 months after treatment and had disappeared at 8 months, but IAPT treatments had cost more per patient, than those provided in neighbouring boroughs.^13^ According to reports compiled by the Artemis Trust,^14,15^ which evaluated data from the subsequent national roll out, the average number of patients achieving recovery for a fixed expenditure of £100 000, when treated by an IAPT service was far lower (49) than for pre-IAPT primary care counselling services (115) or voluntary sector counselling services (78). In addition recovery rates, as a percentage of patients referred, was lower for IAPT services than comparable services (pre-IAPT primary care therapy services, university counselling services and employee assistance programme counselling services).

This is a truly remarkable achievement. The government have spent large amounts of taxpayers’ money creating an expensive service that provides little choice and has poorer outcomes than cheaper alternatives that were already in existence before IAPT. In terms of efficiency this has parallels with many large-scale government contracts, whether in information technology (we all know about the billions wasted trying to link up NHS information technology systems for example), building or procurement, where large amounts are put into monopoly contracts, that overcharge and deliver poor quality products that never quite work the way they were meant to.

Perhaps IAPT has helped reduce medicalisation? Again the answer is No. Prescriptions for antidepressants have continued to rise with little evidence that introducing IAPT has had any meaningful impact on these trajectories. Numbers of people claiming disability living allowance in the UK for a mental health problem has also continued to rise with psychiatric disorders as the reason for receiving disability benefits rising from 30.9% of claimants in 2000 to 44.8% in 2013, with the biggest subcategory (over 50%) being for people given a diagnosis of depression (information retrieved from http://tabulation-tool.dwp.gov.uk). If current national models of mental health service delivery were effective, we would not see this picture of steadily worsening long-term outcomes in parallel with steadily increasing expenditure.

We should again not be surprised by these findings. The technical model locates the challenges and dilemmas that people face in late capitalist neoliberal cultures as happening in people’s heads rather than in the wider contexts of their lived experience. The task of therapies such as CBT is that of helping the person adjust and learn to deal with the pessimistic thoughts that have come to dominate their life. Although I have no problem appreciating the usefulness of this approach for many people, the fetishisation and commodification of ‘suffering’ at the cultural/political level acts to create new markets for ‘treatment’ while simultaneously obscuring the brutal nature of our winner/loser culture through individualising people’s problems. An approach that fails to appreciate the social, cultural and political dimension of distress is thus unlikely to address the problem of the expanding medicalisation of suffering.

## Children and Young People’s IAPT (CYP-IAPT)

As with the adult outcome literature, there is little evidence to support that matching a treatment model to a diagnosis differentiates which treatment is more likely to work and which is not in children and young people.^16,17^ It seems that ‘evidence-based’ treatments for youth tend to come out as superior to usual care, only if the ‘evidence-based’ treatment was developed by the researcher.^18^ Technical factors appear irrelevant. Thus, a meta-analysis of component studies found that the theoretically purported critical ingredients of CBT are not specifically ameliorative for child and adolescent depression and anxiety as full CBT treatments offered no significant benefit over treatments with only components of the full model.^19^

When real-life clinical outcomes from Child and Adolescent Mental Health Services (CAMHS) are examined the picture is even less encouraging. Research has found that 40–60% of youth who begin treatment drop out against advice.^20^ Furthermore, although the effect size for outcomes in controlled studies is large, in traditional treatment in community CAMHS effect sizes are close to zero^21^ with little difference found in outcome between treated and untreated children.^22,23^

Other evidence finds that service transformation projects including allocating extra resources have a negligible impact on outcomes. The Fort Bragg evaluation described the implementation, quality, costs, and outcomes of a $94 million demonstration project designed to improve mental health outcomes for children and adolescents who were referred for mental health treatment. Outcomes in the experimental service were no better than those in the treatment as usual group, despite the considerable extra costs incurred.^24,25^ This finding was then replicated in the Stark County evaluation study where again there were no differences in outcomes when compared with care received outside the new system, despite the extra expenditure.^26^

These are sobering findings suggesting that, just as with adults, traditional, medical/technical model approaches do not appear to provide much ‘added value’ in terms of improving the outcomes and efficiency of services.

### Did the above findings have an impact on the design of the CYP-IAPT project?

In 2011 IAPT gave birth to the CYP-IAPT project. This upstart announced it was going to strike out in a new direction. But like many children who criticise their parents, the values they carry was already part of their histories, and the bold new direction they boasted about amounted to new directions in the scope of implementation without any recognisable change in the underlying paradigm. Indeed, CYP-IAPT decided to start by focusing on improving the skills of the existing CAMHS workforce and to achieve this by training staff in the manualised implementation of CBT or parenting management treatment (in phase 1). As far as the basics go CYP-IAPT was, therefore, no different to its parent IAPT project. However, another and more interesting objective of the CYP-IAPT project was that of ‘service transformation’. Here the plan was to influence the whole CAMHS team to use more feedback-informed approaches including use of session-by-session outcome ratings. Having been involved in a successful ‘service transformation’ project with my own team involving implementing session-by-session outcome monitoring and developing an outcomes database for the team, I was flattered to be invited to join the CYP-IAPT steering group. Perhaps CYP-IAPT was going to go in a new exciting direction after all. My resulting flirtation with CYP-IAPT proved to be a short lived, but fascinating, insight into how bureaucratisation happens when large monolithic programmes are attempted.

Instead of building on existing and successful service transformation projects that have been developed in other countries and in the UK^16^ (and I must declare a potential conflict of interest here – at present ideological rather than financial), the service transformation CYP-IAPT aimed for used the same expensive technological paradigm adhered to by the inefficient IAPT project. The millions given to this programme is being spent on sending CAMHS clinicians to train in the delivery of manualised treatments (such as CBT or parent management). These clinicians’ time then needs to be backfilled, and once trained they are to come back and deliver these therapies in diagnostic-based pathways. A course for managers and extensive implementation checklists have been developed adding greater complexity to service transformation while missing out on learning from whole service projects that have already demonstrated how you might achieve improved outcomes and efficiency. This choking bureaucratisation seems to happen whenever such national projects are attempted in CAMHS.

For example, the CAMHS Outcomes Research Consortium (CORC) has been operating as a UK national project since 2004 with the aim of instituting a common model of routine outcome evaluation and data analysis. However, return rates for second scores on the main patient-rated outcome measure have run at 10–25% or lower for years, thus no reliable and therefore valid outcome data has, at any point, been produced. No matter what they did they could not improve the return rate because the project failed to connect with the reality that front-line clinicians’ face. Such national projects are at constant risk of morphing into ever more complex systems that offer little to help the daily practice of hard-pressed clinicians and therefore little to offer patients.

In my own service we have continued to develop an ‘outcome orientated’ approach^16^ drawing on the successful American ‘Partners for Change Outcome Management Systems’ (PCOMS) model.^27^ Indeed, PCOMS is recognised as an evidence-based model by the USA ‘Substance Abuse and Mental Health Services Administration’ (SAMHSA) National Registry of Evidence-based Programs and Practices on the basis of sufficient randomised controlled trial research. Although it would be insulting and disrespectful to the diversity of opinions in our CAMHS service to claim our project has been a runaway success without immense and problematic aspects, what I can, I believe, claim is that drawing on and building on models that have already demonstrated improved outcomes, improved efficiency, improved recovery rates and decreased medicalisation; has engaged clinicians, proved cheap and efficient and built a database of outcomes for the whole service in under a year. I can tell you my own outcome data for open and discharged cases as well as the outcomes for the team I work with and our service as a whole. We have simple formats that provide our commissioners with the sort of whole-service outcome data they have never previously had. We did not need expensive formulaic trainings, just building on the existing skills of the workforce and providing a feedback mechanism that helps us focus on recovery and enhancing reflective practice.

## Conclusion

The evidence from a variety of outcome studies provides important pointers for how we should design our services. Extra-therapeutic factors are by far the biggest factor influencing outcomes, which should help us have a little more humility about the task of helping people experiencing mental distress. When we deliver services, matching model of treatment to diagnosis is not only a waste of time (given its clinically insignificant impact on outcomes), but fetishising approaches denies patients choice and flexibility, leading to more potential for disengaging from treatment if the model used is not connecting meaningfully for them (a major problem in delivering our Western psychotherapies with marginalised groups such as ethnic minorities). It is clear to me, and an increasing number of psychiatrists, psychologists and researchers that our allegiance to the technical model for understanding mental distress and behavioural deviance is a big mistake. Meaningful transformations in mental healthcare are unlikely to come through projects like IAPT and CYP-IAPT that can not see this. Instead what we get when we go down the technical route is reduced potential patient choice, poor value for money, increasing medicalisation and bureaucracies that alienate clinicians.

I realise that in the face of powerful well-funded organisations, I am powerless to influence meaningful change. But given the overwhelming evidence and so many critics, perhaps together we can foment enough momentum to make possible a more informed national debate to take place that would lead to a more evidence-based approach and future reform of well-intentioned but misguided projects like CYP-IAPT.
